# Analysis of Caputo fractional-order model for COVID-19 with lockdown

**DOI:** 10.1186/s13662-020-02853-0

**Published:** 2020-08-03

**Authors:** Idris Ahmed, Isa Abdullahi Baba, Abdullahi Yusuf, Poom Kumam, Wiyada Kumam

**Affiliations:** 1 KMUTTFixed Point Research Laboratory, Department of Mathematics, Room SCL 802 Fixed Point Laboratory, Science Laboratory Building, Faculty of Science, King Mongkut’s University of Technology Thonburi (KMUTT), 126 Pracha-Uthit Road, Bang Mod, Thung Khru, Bangkok, 10140 Thailand; 2 KMUTT-Fixed Point Theory and Applications Research Group (KMUTT-FPTA), Theoretical and Computational Science Center (TaCS), Science Laboratory Building, Faculty of Science, King Mongkut’s University of Technology Thonburi (KMUTT), 126 Pracha-Uthit Road, Bang Mod, Thung Khru, Bangkok, 10140 Thailand; 3 Department of Mathematics and Computer Science, Sule Lamido University, P.M.B 048, Kafin-Hausa, Jigawa State Nigeria; 4 Department of Mathematical Science, Bayero University Kano, Kano, Nigeria; 5 Department of Computer Engineering, Biruni University, Istanbul, 34010 Turkey; 6 Department of Mathematics, Federal University Dutse, Jigawa, 7156 Nigeria; 7 Program in Applied Statistics, Department of Mathematics and Computer Science, Rajamangala University of Technology Thanyaburi, Thanyaburi, Pathumthani 12110 Thailand

**Keywords:** 47H10, 34A12, 39A30, Lockdown, Coronavirus, Existence and uniqueness, Ulam–Hyers stability, Mathematical model

## Abstract

One of the control measures available that are believed to be the most reliable methods of curbing the spread of coronavirus at the moment if they were to be successfully applied is lockdown. In this paper a mathematical model of fractional order is constructed to study the significance of the lockdown in mitigating the virus spread. The model consists of a system of five nonlinear fractional-order differential equations in the Caputo sense. In addition, existence and uniqueness of solutions for the fractional-order coronavirus model under lockdown are examined via the well-known Schauder and Banach fixed theorems technique, and stability analysis in the context of Ulam–Hyers and generalized Ulam–Hyers criteria is discussed. The well-known and effective numerical scheme called fractional Euler method has been employed to analyze the approximate solution and dynamical behavior of the model under consideration. It is worth noting that, unlike many studies recently conducted, dimensional consistency has been taken into account during the fractionalization process of the classical model.

## Introduction

Coronavirus (COVID-19) pandemic cut across more than 190 countries in the first 20 weeks after its emergence. It started from Wuhan in China, and by mid-March 2020 more than 334,000 people were affected with about 14,500 death cases. The number of positive cases is about 1,210,956 and the number of fatality cases reaches 67,594 by the beginning of April 2020 [1]. It is clear that different people exhibit different behavior regarding the epidemic within the same country and from one country to the other. Mathematical models help in predicting the course of the pandemic and also help in determining why the infection is not uniform [2, 3]. Health systems also use such results as tools in deciding the type of control measures to be adopted. They also help them in deciding the time and places that need applications of the controls. Therefore, it is of paramount importance to understand the transmission dynamics of the disease and to predict whether the control measures available will help in curtailing the spread of the disease [4, 5].

The first case of COVID-19 in Nigeria was recorded on February 27, 2020, in Lagos [6]. Federal Ministry of Health in Nigeria started the course of action by screening travelers and shutting down schools from the mid of March 2020. However, compared to the course of the epidemic in western countries, either the number of asymptomatic cases in Nigeria was higher or the epidemic in Nigeria progressed through a slow phase. In the absence of an effective vaccine for COVID-19 prevention, the only remaining options are prevention of further influx of migrant cases at airports and seaports and contact tracing. China learned from its experience that only complete shut down prevented further spread, and Italy learned from its experience that negligence of communities towards simple public health strategies leads to uncontrolled morbidity and mortality. This prompted the action of the government of Nigeria in imposing 14-day lockdown in some of its states starting on March 30, 2020 [6].

Many studies suggest that from the beginning of coronavirus infection to hospitalization takes the maximum of 10 days, and the incubation period is 2 to 14 days maximum [7, 8]. Also the time between the start of symptomatic manifestations and death is approximately 2–8 weeks [9]. Another study reports that the duration of viral shedding is 8–37 days [10]. A lot of factors help in providing the effectiveness of the intervention strategies, and as recommended by a recent report, it is vital to estimate the optimal periods to implement each intervention [11]. A lot of countries have implemented the strategy of self-quarantine to prevent spread of the virus. Hence, it is of paramount importance to mathematically study the significance of the lockdown in mitigating the spread of COVID-19 with respect to each country, since the contact patterns between individuals are highly non-homogeneous across each population.

It is important to note that, in the classical order model, the state of epidemic model does not depend on its history. However, in real life memory plays a vital role in studying the pattern of spread of any epidemic disease. It was found that the waiting times between doctor visits for a patient follow a power law model [12]. It is worth to know that Caputo fractional time derivative is a consequence of power law [13]. When dealing with real world problem, Caputo fractional-order derivatives allow traditional initial and boundary conditions. Furthermore, due to its nonlocal behavior and its ability to change at every instant of time, Caputo fractional order gives better result than the integer order [14–20].

In recent studies, Khan et al. [21] studied a fractional-order model that describes the interaction among bats and unknown hosts, then among people and seafood market. To predict the trend of the coronavirus Yu et al. [22] constructed a fractional time delay dynamic system that studied the local outbreak of COVID-19. Also, to predict the possible outbreak of infectious diseases like COVID-19 and other diseases in the future, Xu et al. [23] proposed a generalized fractional-order SEIQRD model. Shaikh et al. [24] used bats-hosts-reservoir-people transmission fractional-order COVID-19 model to estimate the effectiveness of preventive measures and various mitigation strategies, predicting future outbreaks and potential control strategies. Many models in the literature studied the dynamics of COVID-19, some predicted the number of susceptible individuals in a given community, but none of them to our knowledge studied the significance or otherwise of the embattled lockdown. Also most of these models were integer-order models.

The main aim of this research is to study a fractional-order epidemic model that investigates the significance of lockdown in mitigating the spread of COVID-19. Based on the memorability nature of Caputo fractional-order derivatives, this model can be fitted with data reasonably well. Then, based on the official data given by NCDC every day, several numerical examples are exhibited to verify the rationality of the fractional-order model and the effectiveness of the lockdown. Compared with an integer-order system ($\upsilon = 1$), the fractional-order model without network is validated to have a better fitting of the data on Nigeria.

This paper is organized as follows. In Sect. 2, preliminary definitions are given. In Sect. 3, the fractional-order model for COVID-19 in the Caputo sense is formulated. In Sect. 4, existence and uniqueness of the solution of the model is established. In Sect. 5, stability analysis of the solution of the model in the frame of Ulam–Hyers and generalized Ulam–Hyers is given. Section 6 contains the numerical scheme and numerical simulations to illustrate the theoretical results. Finally, conclusion is given in Sect. 7.

## Preliminaries

### Definition 1

([25])

Suppose $\upsilon >0$ and $g\in L^{1}([0, b], \mathbb{R})$ where $[0, b]\subset \mathbb{R}_{+}$. Then fractional integral of order *υ* for a function *g* in the sense of Riemann–Liouville is defined as

$$ \mathcal{I}^{\upsilon }_{0^{+}}g(t)=\frac{1}{\varGamma (\upsilon )} \int _{0}^{t}(t- \tau )^{\upsilon -1}g(\tau )\,d\tau , \quad t>0, $$

where $\varGamma (\cdot )$ is the classical gamma function defined by

1 $$ \varGamma (\upsilon )= \int _{0}^{\infty }\tau ^{\upsilon -1}e^{-\tau }\,d \tau . $$

### Definition 2

([25])

Let $n-1<\upsilon <n$, $n\in \mathbb{N}$, and $g\in C^{n}[0, b]$. The Caputo fractional derivative of order *υ* for a function *g* is defined as

$$ {}^{C}\mathcal{D}^{\upsilon }_{0+}g(t)=\frac{1}{\varGamma (n-\upsilon )} \int _{0}^{t}(t-\tau )^{n-\upsilon -1}g^{n}( \tau )\,d\tau , \quad t>0. $$

### Lemma 1

([25])

*Let*$\operatorname{Re}(\upsilon )>0$, $n=[\operatorname{Re}(\upsilon )]+1$, *and*$g\in AC^{n}(0, b)$. *Then*

2 $$ \bigl({\mathcal{J}_{0^{+}}^{\upsilon }} {}^{C} \mathcal{D}_{0^{+}}^{\upsilon }g\bigr) (t)=g(t)- \sum _{k=1}^{m}\frac{(D_{0^{+}}^{k}g)(0^{+})}{k!}t^{k}. $$

*In particular*, *if*$0<\upsilon \leq 1$, *then*

3 $$ \bigl({\mathcal{J}_{0^{+}}^{\upsilon }} {}^{C} \mathcal{D}_{0^{+}}^{\upsilon }g\bigr) (t)=g(t)-g_{0}. $$

## Formulation of the model

Let the total population be $N(t)$. The population is divided into four compartments, namely susceptible population that are not under lockdown $S(t)$, susceptible population that are under lockdown $S_{L} (t)$, infective population that are not under lockdown $I(t)$ (here we refer to isolation as lockdown for convenience), infective population that are under lockdown $I_{L}(t)$, and then cumulative density of the lockdown program $L(t)$. The dynamics of this population is represented by the following system of fractional-order differential equations (FODE), and the meaning of parameters is given in Table 1.

4 $$ \textstyle\begin{cases} {}^{C}\mathcal{D}^{\upsilon }_{0^{+}}S(t)=\varLambda^{\upsilon} -\beta^{\upsilon} S I-\lambda^{\upsilon} _{1}SL- \bar{d}^{\upsilon}S+\gamma^{\upsilon} _{1}I+\gamma^{\upsilon} _{2}I_{L}+\theta^{\upsilon} _{1}S_{L},&\\ {}^{C}\mathcal{D}^{\upsilon }_{0^{+}}S_{L}(t)=\lambda^{\upsilon} _{1}SL-\bar{d}^{\upsilon}S_{L}- \theta _{1}^{\upsilon}S_{L}, \\ {}^{C}\mathcal{D}^{\upsilon }_{0^{+}}I(t)=\beta^{\upsilon} S I-\gamma^{\upsilon} _{1}I-\alpha^{\upsilon} _{1}I- \bar{d}^{\upsilon}I+\lambda^{\upsilon} _{2}I L+\theta^{\upsilon} _{2}I_{L}, \\ {}^{C}\mathcal{D}^{\upsilon }_{0^{+}}I_{L}(t)=\lambda^{\upsilon} _{2}I L-\bar{d}^{\upsilon}I_{L}- \theta^{\upsilon} _{2}I_{L}-\gamma^{\upsilon} _{2}I_{L}-\alpha^{\upsilon} _{2}I_{L}, \\ {}^{C}\mathcal{D}^{\upsilon }_{0^{+}}L(t)=\mu^{\upsilon} I-\phi^{\upsilon} L. \end{cases} $$

**Table 1:** Description of the parameters

Parameter	Description
*Λ*	recruitment rate
*β*	infection contact rate
$\lambda _{1}$, $\lambda _{2}$	imposition of lockdown on susceptible and infectives respectively
$\gamma _{1}$, $\gamma _{2}$	recovery rate in *I* and $I_{L}$ respectively
$\alpha _{1}$, $\alpha _{2}$	death rate due to infection in *I* and $I_{L}$ respectively
*d̄*	natural death rate
$\theta _{1} $	rate of transfer of susceptible lockdown individuals to susceptible class
$\theta _{2} $	rate of transfer of infective lockdown individuals to infective class
*μ*	rate of implementation of the lockdown program
*ϕ*	rate of depletion of the lockdown program

## Existence and uniqueness results

The theory of existence and uniqueness of solutions is one of the most dominant fields in the theory of fractional-order differential equations. The theory has recently attracted the attention of many researchers, we are referring to [26–31] and the references therein for some of the recent growth. In this section, we discuss the existence and uniqueness of solutions of the proposed model using fixed point theorems. We simplify the proposed model (4) in the following setting:

5 $$ \textstyle\begin{cases} {}^{C}\mathcal{D}^{\upsilon }_{0^{+}}S(t)=\varTheta _{1}(t, S, S_{L}, I, I_{L}, L),&\\ {}^{C}\mathcal{D}^{\upsilon }_{0^{+}}S_{L}(t)=\varTheta _{2}(t, S, S_{L}, I, I_{L}, L), \\ {}^{C}\mathcal{D}^{\upsilon }_{0^{+}}I(t)=\varTheta _{3}(t, S, S_{L}, I, I_{L}, L), \\ {}^{C}\mathcal{D}^{\upsilon }_{0^{+}}I_{L}(t)=\varTheta _{4}(t, S, S_{L}, I, I_{L}, L), \\ {}^{C}\mathcal{D}^{\upsilon }_{0^{+}}L(t)=\varTheta _{5}(t, S, S_{L}, I, I_{L}, L), \end{cases} $$

where

6 $$ \textstyle\begin{cases} \varTheta _{1}(t, S, S_{L}, I, I_{L}, L)=\varLambda^{\upsilon } -\beta^{\upsilon } S I-\lambda^{\upsilon } _{1}SL- \bar{d}^{\upsilon }S+\gamma^{\upsilon } _{1}I+\gamma^{\upsilon } _{2}I_{L}+\theta^{\upsilon } _{1}S_{L},&\\ \varTheta _{2}(t, S, S_{L}, I, I_{L}, L)=\lambda^{\upsilon } _{1}SL-\bar{d}^{\upsilon }S_{L}- \theta^{\upsilon } _{1}S_{L}, \\ \varTheta _{3}(t, S, S_{L}, I, I_{L}, L)=\beta^{\upsilon } S I-\gamma^{\upsilon } _{1}I-\alpha^{\upsilon } _{1}I- \bar{d}^{\upsilon }I+\lambda^{\upsilon } _{2}I L+\theta^{\upsilon } _{2}I_{L}, \\ \varTheta _{4}(t, S, S_{L}, I, I_{L}, L)=\lambda^{\upsilon } _{2}I L-\bar{d}^{\upsilon }I_{L}- \theta^{\upsilon } _{2}I_{L}-\gamma^{\upsilon } _{2}I_{L}-\alpha^{\upsilon } _{2}I_{L}, \\ \varTheta _{5}(t, S, S_{L}, I, I_{L}, L)=\mu^{\upsilon } I-\phi^{\upsilon } L. \end{cases} $$

Thus, the proposed model (4) takes the form

7 $$ \textstyle\begin{cases} {}^{C}\mathcal{D}^{\alpha }_{0}\varPhi (t)=\mathcal{K}(t, \varPhi (t)),\quad t \in J=[0, b],0< \alpha \le 1,&\\ \varPhi (0)=\varPhi _{0}\ge 0, \end{cases} $$

on condition that

8 $$ \textstyle\begin{cases} \varPhi (t)=(S, S_{L}, I, I_{L}, L)^{T},&\\ \varPhi (0)=(S_{0}, {S_{L}}_{0}, I_{0}, {I_{L}}_{0}, L_{0})^{T}, \\ \mathcal{K}(t, \varPhi (t))=(\varTheta _{i}(t, S, S_{L}, I, I_{L}, L))^{T},\quad i=1,\ldots,5, \end{cases} $$

where $(\cdot )^{T}$ represents the transpose operation. In view of Lemma 1, the integral representation of problem (7) which is equivalent to model (4) is given by

9 $$ \begin{aligned}[b] \varPhi (t)&=\varPhi _{0}+ \mathcal{J}_{0^{+}}^{\upsilon } \mathcal{K}\bigl(t, \varPhi (t)\bigr) \\ &=\varPhi _{0}+\frac{1}{\varGamma (\upsilon )} \int _{0}^{t}(t-\tau )^{ \upsilon -1}\mathcal{K} \bigl(\tau , \varPhi (\tau )\bigr)\,d\tau . \end{aligned} $$

Let $\mathbb{E}= C([0, b]; \mathbb{R})$ denote the Banach space of all continuous functions from $[0, b]$ to $\mathbb{R}$ endowed with the norm defined by

$$ \Vert \varPhi \Vert _{\mathbb{E}}= \sup_{t\in J} \bigl\vert \varPhi (t) \bigr\vert , $$

where

$$ \bigl\vert \varPhi (t) \bigr\vert = \bigl\vert S(t) \bigr\vert + \bigl\vert S_{L}(t) \bigr\vert + \bigl\vert I(t) \bigr\vert + \vert I_{L} \vert + \bigl\vert L(t) \bigr\vert , $$

and $S,S_{L},I,I_{L},L\in C([0, b], \mathbb{R})$.

### Theorem 1

*Suppose that the function*$\mathcal{K}\in C([J, \mathbb{R}])$*and maps a bounded subset of*$J\times \mathbb{R}^{5}$*into relatively compact subsets of*$\mathbb{R}$. *In addition*, *there exists constant*$\mathcal{L}_{\mathcal{K}}>0$*such that*

$(A_{1})$
$\vert \mathcal{K}(t, \varPhi _{1}(t))-\mathcal{K}(t, \varPhi _{2}(t)) \vert \le \mathcal{L}_{\mathcal{K}} \vert \varPhi _{1}(t)-\varPhi _{2}(t) \vert $*for all*$t\in J$*and each*$\varPhi _{1},\varPhi _{2}\in C([J, \mathbb{R}])$. *Then problem* (7) *which is equivalent to the proposed model* (4) *has a unique solution provided that*$\varOmega \mathcal{L}_{\mathcal{K}}<1$, *where*
  $$ \varOmega =\frac{b^{\upsilon }}{\varGamma (\upsilon +1)}. $$

### Proof

Consider the operator $P : \mathbb{E}\rightarrow \mathbb{E}$ defined by

10 $$ (P\varPhi ) (t)=\varPhi _{0}+\frac{1}{\varGamma (\upsilon )} \int _{0}^{t}(t- \tau )^{\upsilon -1}\mathcal{K} \bigl(\tau , \varPhi (\tau )\bigr)\,d\tau . $$

Obviously, the operator *P* is well defined and the unique solution of model (4) is just the fixed point of *P*. Indeed, let us take $\sup_{t\in J} \Vert \mathcal{K}(t, 0) \Vert =M_{1}$ and $\kappa \ge \Vert \varPhi _{0} \Vert +\varOmega M_{1}$. Thus, it is enough to show that $P\mathbb{B}_{\kappa }\subset \mathbb{B}_{\kappa }$, where the set $\mathbb{B}_{\kappa }=\{\varPhi \in \mathbb{E}: \Vert \varPhi \Vert \le \kappa \}$ is closed and convex. Now, for any $\varPhi \in \mathbb{B}_{\kappa }$, it yields

11 $$ \begin{aligned}[b] \bigl\vert (P\varPhi ) (t) \bigr\vert &\le \vert \varPhi _{0} \vert + \frac{1}{\varGamma (\upsilon )} \int _{0}^{t}(t-\tau )^{\upsilon -1} \bigl\vert \mathcal{K}\bigl(\tau , \varPhi (\tau )\bigr) \bigr\vert \,d\tau \\ &\le \varPhi _{0}+\frac{1}{\varGamma (\upsilon )} \int _{0}^{t}(t-\tau )^{ \upsilon -1} \bigl[ \bigl\vert \mathcal{K}\bigl(\tau , \varPhi (\tau )\bigr)-\mathcal{K}( \tau , 0) \bigr\vert + \bigl\vert \mathcal{K}(\tau , 0) \bigr\vert \bigr] \,d\tau \\ &\le \varPhi _{0}+ \frac{(\mathcal{L}_{\mathcal{K}}\kappa +M_{1})}{\varGamma (\upsilon )} \int _{0}^{t}(t-\tau )^{\upsilon -1}\,d\tau \\ &\le \varPhi _{0}+ \frac{(\mathcal{L}_{\mathcal{K}}\kappa +M_{1})}{\varGamma (\upsilon +1)}b^{ \upsilon } \\ &\le \varPhi _{0}+\varOmega (\mathcal{L}_{\mathcal{K}}\kappa +M_{1}) \\ &\le \kappa . \end{aligned} $$

Hence, the results follow. Also, given any $\varPhi _{1},\varPhi _{2}\in \mathbb{E}$, we get

12 $$ \begin{aligned}[b] \bigl\vert (P\varPhi _{1}) (t)-(P\varPhi _{2}) (t) \bigr\vert &\le \frac{1}{\varGamma (\upsilon )} \int _{0}^{t}(t-\tau )^{\upsilon -1} \bigl\vert \mathcal{K}\bigl(\tau , \varPhi _{1}(\tau )\bigr)-\mathcal{K}\bigl(\tau , \varPhi _{2}( \tau )\bigr) \bigr\vert \,d\tau \\ &\le \frac{\mathcal{L}_{\mathcal{K}}}{\varGamma (\upsilon )} \int _{0}^{t}(t- \tau )^{\upsilon -1} \bigl\vert \varPhi _{1}(\tau )-\varPhi _{2}(\tau ) \bigr\vert \,d\tau \\ &\le \varOmega \mathcal{L}_{\mathcal{K}} \bigl\vert \varPhi _{1}(t)-\varPhi _{2}(t) \bigr\vert , \end{aligned} $$

which implies that $\Vert (P\varPhi _{1})-(P\varPhi _{2}) \Vert \le \varOmega \mathcal{L}_{\mathcal{K}} \Vert \varPhi _{1}-\varPhi _{2} \Vert $. Therefore, as a consequence of the Banach contraction principle, the proposed model (4) has a unique solution. □

Next, we prove the existence of solutions of problem (7) which is equivalent to the proposed model (4) by employing the concept of Schauder fixed point theorem. Thus, the following assumption is needed.

$(A_{2})$
Suppose that there exist $\sigma _{1},\sigma _{2}\in \mathbb{E}$ such that
  $$ \bigl\vert \mathcal{K}\bigl(t, \varPhi (t)\bigr) \bigr\vert \le \sigma _{1}(t)+\sigma _{2} \bigl\vert \varPhi (t) \bigr\vert \quad \text{for any }\varPhi \in \mathbb{E}, t\in J, $$
  such that $\sigma _{1}^{*}=\sup_{t\in J} \vert \sigma _{1}(t) \vert $, $\sigma _{2}^{*}= \sup_{t\in J} \vert \sigma _{2}(t) \vert <1$.

### Lemma 2

*The operatorPdefined in* (10) *is completely continuous*.

### Proof

Obviously, the continuity of the function $\mathcal{K}$ gives the continuity of the operator *P*. Thus, for any $\varPhi \in \mathbb{B}_{\kappa }$, where $\mathbb{B}_{\kappa }$ is defined above, we get

13 $$\begin{aligned} \bigl\vert (P\varPhi ) (t) \bigr\vert &= \biggl\vert \varPhi _{0}+ \frac{1}{\varGamma (\upsilon )} \int _{0}^{t}(t-\tau )^{\upsilon -1} \mathcal{K} \bigl(\tau , \varPhi (\tau )\bigr)\,d\tau \biggr\vert \\ &\le \Vert \varPhi _{0} \Vert +\frac{1}{\varGamma (\upsilon )} \int _{0}^{t}(t-\tau )^{ \upsilon -1} \bigl\vert \mathcal{K}\bigl(\tau , \varPhi (\tau )\bigr) \bigr\vert \,d\tau \\ &\le \Vert \varPhi _{0} \Vert + \frac{(\sigma _{1}^{*}+\sigma _{2}^{*} \Vert \varPhi \Vert )}{\varGamma (\upsilon )} \int _{0}^{t}(t-\tau )^{\upsilon -1}\,d\tau \\ &\le \Vert \varPhi _{0} \Vert + \frac{(\sigma _{1}^{*}+\sigma _{2}^{*} \Vert \varPhi \Vert )}{\varGamma (\upsilon +1)}b^{ \upsilon } \\ &= \Vert \varPhi _{0} \Vert +\varOmega \bigl(\sigma _{1}^{*}+\sigma _{2}^{*} \Vert \varPhi \Vert \bigr) \\ &< +\infty . \end{aligned}$$

So, the operator *P* is uniformly bounded. Next, we prove the equicontinuity of *P*. To do so, we let $\sup_{(t, \varPhi )\in J\times \mathbf{B}_{\kappa }} \vert \mathcal{K}(t, \varPhi (t)) \vert =\mathcal{K}^{*}$. Then, for any $t_{1},t_{2}\in J$ such that $t_{2}\ge t_{1}$, it gives

14 $$ \begin{aligned}[b] \bigl\vert (P\varPhi ) (t_{2})-(P\varPhi ) (t_{1}) \bigr\vert &= \frac{1}{\varGamma (\upsilon )} \biggl\vert \int _{0}^{t_{1}}\bigl[(t_{2}-\tau )^{ \upsilon -1}-(t_{1}-\tau )^{\upsilon -1}\bigr]\mathcal{K}\bigl(\tau , \varPhi ( \tau )\bigr)\,d\tau \\ &\quad{} + \int _{t_{1}}^{t_{2}}(t_{2}-\tau )^{\upsilon -1} \mathcal{K}\bigl(\tau , \varPhi (\tau )\bigr)\,d\tau \biggr\vert \\ &\le \frac{\mathcal{K}^{*}}{\varGamma (\upsilon )} \bigl[2(t_{2}-t_{1})^{ \upsilon }+ \bigl(t_{2}^{\upsilon }-t_{1}^{\upsilon }\bigr) \bigr] \\ &\rightarrow 0,\quad \text{as } t_{2}\rightarrow t_{1}. \end{aligned} $$

Hence, the operator *P* is equicontinuous and so is relatively compact on $\mathbf{B}_{\kappa }$. Therefore, as a consequence of Arzelá–Ascoli theorem, *P* is completely continuous. □

### Theorem 2

*Suppose that the function*$\mathcal{K}:J\times \mathbb{R}^{5}\rightarrow \mathbb{R}$*is continuous and satisfies assumption*$(A_{2})$. *Then problem* (7) *which is equivalent with the proposed model* (4) *has at least one solution*.

### Proof

We define a set $\mathcal{U}=\{\varPhi \in \mathbb{E}: \varPhi =o(P\varPhi )(t), 0< o<1\}$. Clearly, in view of Lemma 2, the operator $P: \mathcal{U}\rightarrow \mathbb{E}$ as defined in (10) is completely continuous. Now, for any $\varPhi \in \mathcal{U}$ and assumption $(A_{2})$, it yields

15 $$ \begin{aligned}[b] \bigl\vert (\varPhi ) (t) \bigr\vert &= \bigl\vert o(P\varPhi ) (t) \bigr\vert \\ &\le \vert \varPhi _{0} \vert +\frac{1}{\varGamma (\upsilon )} \int _{0}^{t}(t-\tau )^{ \upsilon -1} \bigl\vert \mathcal{K}\bigl(\tau , \varPhi (\tau )\bigr) \bigr\vert \,d\tau \\ &\le \Vert \varPhi _{0} \Vert + \frac{(\sigma _{1}^{*}+\sigma _{2}^{*} \Vert \varPhi \Vert )}{\varGamma (\upsilon +1)}b^{ \upsilon } \\ &= \Vert \varPhi _{0} \Vert +\varOmega \bigl(\sigma _{1}^{*}+\sigma _{2}^{*} \Vert \varPhi \Vert \bigr) \\ &< +\infty . \end{aligned} $$

Thus, the set $\mathcal{U}$ is bounded. So the operator *P* has at least one fixed point which is just the solution of the proposed model (4). Hence the desired result. □

## Stability results

In this section, we derive the stability of the proposed model (4) in the frame of Ulam–Hyers and generalized Ulam–Hyers stability. The concept of Ulam stability was introduced by Ulam [32, 33]. Then, in several research papers on classical fractional derivatives, the aforementioned stability was investigated, see for example [34–38]. Moreover, since stability is fundamental for approximate solution, we strive to use nonlinear functional analysis on Ulam–Hyers and generalized stability of the proposed model (4). Thus the following definitions are needed. Let $\epsilon >0$ and consider the following inequality:

16 $$ \bigl\vert {}^{C}\mathcal{D}_{0^{+}}^{\upsilon } \bar{\varPhi }(t)-\mathcal{K}\bigl(t, \bar{\varPhi }(t)\bigr) \bigr\vert \leq \epsilon , \quad t\in J, $$

where $\epsilon =\max (\epsilon _{j})^{T}$, $j=1,\ldots,5$.

### Definition 3

The proposed problem (7), which is equivalent to model (4), is Ulam–Hyers stable if there exists $\mathcal{C}_{\mathcal{K}}>0$ such that, for every $\epsilon >0$ and for each solution $\bar{\varPhi }\in \mathbb{E}$ satisfying inequality (3), there exist a solution $\varPhi \in \mathbb{E}$ of problem (7), with

$$ \bigl\vert \bar{\varPhi }(t)-\varPhi (t) \bigr\vert \leq \mathcal{C}_{\mathcal{K}}\epsilon , \quad t\in J, $$

where $\mathcal{C}_{\mathcal{K}}=\max (\mathcal{C}_{\mathcal{K}_{j}})^{T}$.

### Definition 4

Problem (7), which is equivalent to model (4), is referred to as being generalized Ulam–Hyers stable if there exists a continuous function $\varphi _{\mathcal{K}}: \mathbb{R}_{+} \rightarrow \mathbb{R}_{+}$, with $\varphi _{\mathcal{K}}(0)=0$, such that, for each solution $\bar{\varPhi }\in \mathbb{E}$ of the inequality (16), there is exist a solution $\varPhi \in \mathbb{E}$ of problem (7) such that

$$ \bigl\vert \bar{\varPhi }(t)-\varPhi (t) \bigr\vert \leq \varphi _{\mathcal{K}}\epsilon , \quad t \in J, $$

where $\varphi _{\mathcal{K}}=\max (\varphi _{\mathcal{K}_{j}})^{T}$.

### Remark 1

A function $\bar{\varPhi }\in \mathbb{E}$ is a solution of the inequality (16) if and only if there exist a function $h\in \mathbb{E}$ with the following property:

(i) $\vert h(t) \vert \leq \epsilon $, $h=\max (h_{j})^{T}$, $t\in J$;
(ii) ${}^{C}\mathcal{D}_{0^{+}}^{\upsilon }\bar{\varPhi }(t)=\mathcal{K}(t, \bar{\varPhi }(t))+h(t)$, $t\in J$.

### Lemma 3

*Assume that*$\bar{\varPhi }\in \mathbb{E}$*satisfies inequality* (16), *thenΦ̄satisfies the integral inequality described by*

17 $$ \biggl\vert \bar{\varPhi }(t)-\bar{\varPhi }_{0}-\frac{1}{\varGamma (\upsilon )} \int _{0}^{t}(t- \tau )^{\upsilon -1}\mathcal{K} \bigl(\tau , \bar{\varPhi }(\tau )\bigr)\,d\tau \biggr\vert \leq \varOmega \epsilon . $$

### Proof

Thanks to (ii) of Remark 1,

$$ {}^{C}\mathcal{D}_{0^{+}}^{\upsilon }\bar{\varPhi }(t)= \mathcal{K}\bigl(t, \bar{\varPhi }(t)\bigr)+h(t), $$

and Lemma 1 gives

18 $$ \bar{\varPhi }(t)=\bar{\varPhi }_{0}+\frac{1}{\varGamma (\upsilon )} \int _{0}^{t}(t- \tau )^{\upsilon -1}\mathcal{K} \bigl(\tau , \bar{\varPhi }(\tau )\bigr)\,d\tau + \frac{1}{\varGamma (\upsilon )} \int _{0}^{t}(t-\tau )^{\upsilon -1}h( \tau )\,d\tau . $$

Using $(i)$ of Remark 1, we get

19 $$ \begin{aligned}[b] \biggl\vert \bar{\varPhi }(t)-\bar{\varPhi }_{0}- \frac{1}{\varGamma (\upsilon )} \int _{0}^{t}(t-\tau )^{\upsilon -1} \mathcal{K} \bigl(\tau , \bar{\varPhi }(\tau )\bigr)\,d\tau \biggr\vert &\le \frac{1}{\varGamma (\upsilon )} \int _{0}^{t}(t-\tau )^{\upsilon -1} \bigl\vert h( \tau ) \bigr\vert \,d\tau \\ &\leq \varOmega \epsilon . \end{aligned} $$

Hence, the desired results. □

### Theorem 3

*Suppose that*$\mathcal{K}: J\times \mathbb{R}^{5}\rightarrow \mathbb{R}$*is continuous for every*$\varPhi \in \mathbb{E}$*and assumption*$(A_{1})$*holds with*$1-\varOmega \mathcal{L}_{\mathcal{K}}>0$. *Thus*, *problem* (7) *which is equivalent to model* (4) *is Ulam–Hyers and*, *consequently*, *generalized Ulam–Hyers stable*.

### Proof

Suppose that $\bar{\varPhi }\in \mathbb{E}$ satisfies inequality (16) and $\varPhi \in \mathbb{E}$ is a unique solution of problem (7). Thus, for any $\epsilon >0$, $t\in J$ and Lemma 3, it gives

$$ \begin{aligned} \bigl\vert \bar{\varPhi }(t)-\varPhi (t) \bigr\vert &= \max_{t\in \mathcal{J}} \biggl\vert \bar{\varPhi }(t)-\varPhi _{0}- \frac{1}{\varGamma (\upsilon )} \int _{0}^{t}(t-\tau )^{\upsilon -1} \mathcal{K} \bigl(\tau , \varPhi (\tau )\bigr)\,d\tau \biggr\vert \\ &\le \max_{t\in \mathcal{J}} \biggl\vert \bar{\varPhi }(t)- \bar{\varPhi }_{0}-\frac{1}{\varGamma (\upsilon )} \int _{0}^{t}(t-\tau )^{ \upsilon -1}\mathcal{K} \bigl(\tau , \bar{\varPhi }(\tau )\bigr)\,d\tau \biggr\vert \\ &\quad {}+ \max_{t\in \mathcal{J}}\frac{1}{\varGamma (\upsilon )} \int _{0}^{t}(t-\tau )^{\upsilon -1} \bigl\vert \mathcal{K}\bigl(\tau , \bar{\varPhi }( \tau )\bigr)-\mathcal{K}\bigl(\tau , \varPhi (\tau )\bigr) \bigr\vert \,d\tau \\ &\leq \biggl\vert \varPhi (t)-\bar{\varPhi }_{0}-\frac{1}{\varGamma (\upsilon )} \int _{0}^{t}(t- \tau )^{\upsilon -1}\mathcal{K} \bigl(\tau , \bar{\varPhi }(\tau )\bigr)\,d\tau \biggr\vert \\ &\quad{} +\frac{\mathcal{L}_{\mathcal{K}}}{\varGamma (\upsilon )} \int _{0}^{t}(t- \tau )^{\upsilon -1} \bigl\vert \bar{\varPhi }(\tau )-\varPhi (\tau ) \bigr\vert \,d\tau \\ &\leq \varOmega \epsilon +\varOmega \mathcal{L}_{\mathcal{K}} \bigl\vert \bar{\varPhi }(t)- \varPhi (t) \bigr\vert . \end{aligned} $$

So,

$$ \Vert \bar{\varPhi }-\varPhi \Vert \leq \mathcal{C}_{\mathcal{K}}\epsilon , $$

where

$$ \mathcal{C}_{\mathcal{K}}= \frac{\varOmega }{1-\varOmega \mathcal{L}_{\mathcal{K}}}. $$

Setting $\varphi _{\mathcal{K}}(\epsilon )=\mathcal{C}_{\mathcal{K}}\epsilon $ such that $\varphi _{\mathcal{K}}(0)=0$, we conclude that the proposed problem (4) is both Ulam–Hyers and generalized Ulam–Hyers stable. □

## Numerical simulations and discussion

Herein, the fractional variant of the model under consideration via Caputo fractional operator is numerically simulated via first-order convergent numerical techniques as proposed in [39–41]. These numerical techniques are accurate, conditionally stable, and convergent for solving fractional-order both linear and nonlinear systems of ordinary differential equations.

Consider a general Cauchy problem of fractional order having autonomous nature

20 $$ {}^{\star}D^{\upsilon }_{0,t} \bigl(y(t) \bigr)=g \bigl(y(t) \bigr), \quad \upsilon \in (0,1], t\in [0,T], y(0)=y_{0}, $$

where $y=(a,b,c,w) \in \mathbb{R}^{4}_{+}$ is a real-valued continuous vector function which satisfies the Lipschitz criterion given as

21 $$ \bigl\Vert g\bigl(y_{1}(t)\bigr)-g\bigl(y_{2}(t)\bigr) \bigr\Vert \leq M \bigl\Vert y_{1}(t)-y_{2}(t) \bigr\Vert , $$

where *M* is a positive real Lipschitz constant.

Using the fractional-order integral operators, one obtains

22 $$ y(t)=y_{0}+J^{\upsilon }_{0,t} g\bigl(y(t)\bigr), \quad t \in [0, T], $$

where $J^{\varOmega }_{0,t}$ is the fractional-order integral operator in Riemann–Liouville. Consider an equi-spaced integration intervals over $[0,T]$ with the fixed step size *h* (=10^−2^ for simulation) = $\frac{T}{n}$, $n\in \mathbb{N}$. Suppose that $y_{q}$ is the approximation of $y(t)$ at $t=t_{q}$ for $q=0,1,\ldots, n$. The numerical technique for the governing model under Caputo fractional derivative operator takes the form

23 $$\begin{aligned} \begin{gathered} \begin{aligned} {}^{\mathrm{c}}S_{p+1}={}&a_{0}+ \frac{h^{\upsilon }}{\varGamma (\upsilon +1)} \\ &{}\times\sum_{k=0}^{p} \bigl((p-k+1)^{ \upsilon }-(p-k)^{\upsilon } \bigr) (\varLambda -\beta SI- \lambda _{1}SL- \bar{d}S+\gamma _{1}I+\gamma _{2}I_{L}+\theta _{1}S_{L} ) , \end{aligned} \\ {}^{\mathrm{c}}SL_{p+1}=b_{0}+ \frac{h^{\upsilon }}{\varGamma (\upsilon +1)} \sum _{k=0}^{p} \bigl((p-k+1)^{ \upsilon }-(p-k)^{\upsilon } \bigr) (\lambda _{1}SL-\bar{d}S_{L}- \theta _{1}S_{L} ), \\ {}^{\mathrm{c}}I_{p+1}=d_{0}+ \frac{h^{\upsilon }}{\varGamma (\upsilon +1)} \sum _{k=0}^{p} \bigl((p-k+1)^{ \upsilon }-(p-k)^{\upsilon } \bigr) (\beta SI-\gamma _{1}I-\alpha _{1}I- \bar{d}I+\lambda _{2}IL+\theta _{2}I_{L} ), \\ {}^{\mathrm{c}}IL_{p+1}=e_{0}+ \frac{h^{\upsilon }}{\varGamma (\upsilon +1)} \sum _{k=0}^{p} \bigl((p-k+1)^{ \upsilon }-(p-k)^{\upsilon } \bigr) (\lambda _{2}IL-\bar{d}I_{L}- \theta _{2}I_{L}-\gamma _{2}I_{L}-\alpha _{2}I_{L} ) , \\ {}^{\mathrm{c}}L_{p+1}=f_{0}+ \frac{h^{\upsilon }}{\varGamma (\upsilon +1)} \sum _{k=0}^{p} \bigl((p-k+1)^{ \upsilon }-(p-k)^{\upsilon } \bigr) (\mu I-\phi L ). \end{gathered} \end{aligned}$$

Now we discuss the obtained numerical outcomes of the governing model in respect of the approximate solutions. To this aim, we employed the effective Euler method under the Caputo fractional operator to do the job. The initial conditions are assumed as $S(0)=900$, $S_{L}(0)=300$, $I(0)=300$, $I_{L}(0)=497$, $L(0)=200$ and the parameter values are taken as in Table 2.

**Table 2:** Parameter values

Parameters	Values	References
*Λ*	400	[42]
*β*	0.000017	[43]
$\lambda _{1}$	0.0002	[42]
$\lambda _{2}$	0.002	Assumed
$\gamma _{1}$	0.16979	[6]
$\gamma _{2}$	0.16979	[6]
$\alpha _{1}$	0.03275	[6]
$\alpha _{2}$	0.03275	[6]
*d*	0.0096	[44]
$\theta _{1}$	0.2	[42]
$\theta _{2}$	0.02	Assumed
*μ*	0.0005	[42]
*ϕ*	0.06	[42]

**Figure 1:**
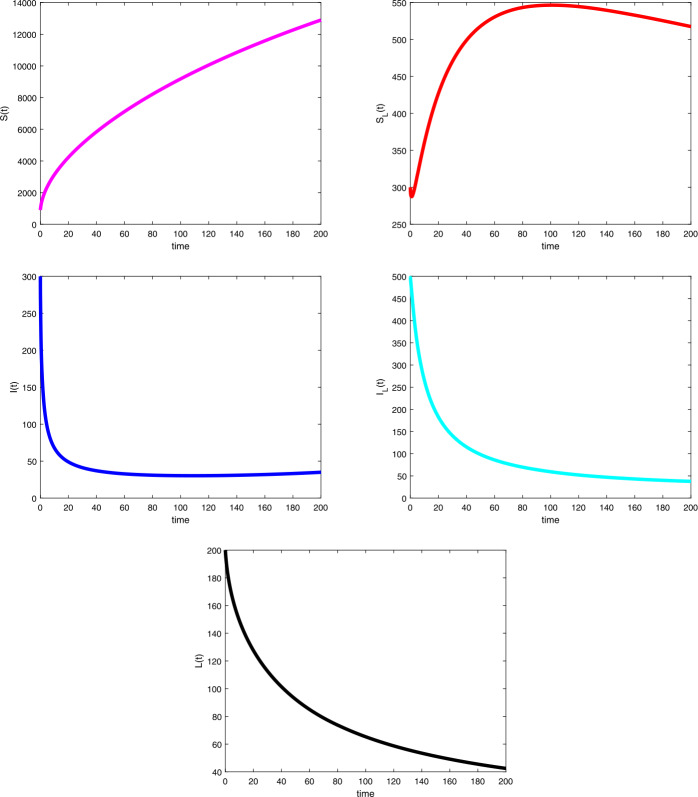
Profiles for behavior of each state variable for the Caputo version of the fractional model using the values of the parameters

**Figure 2:**
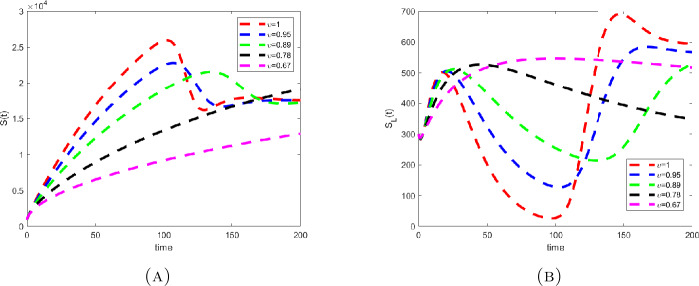
Dynamical outlook of the susceptible class that aren’t under lockdown abd susceptible class that are under lockdown with different fractional-order values

**Figure 3:**
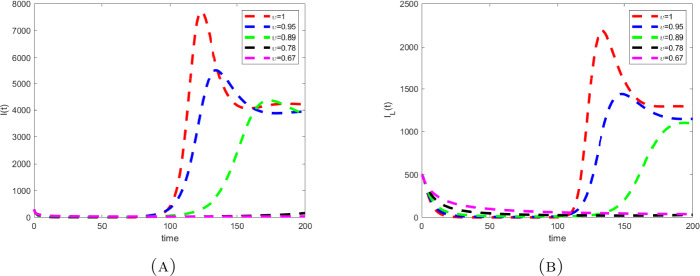
Dynamical outlook of the infective class that aren’t under lockdown and infective class that are under lockdown with different fractional-order values

**Figure 4:**
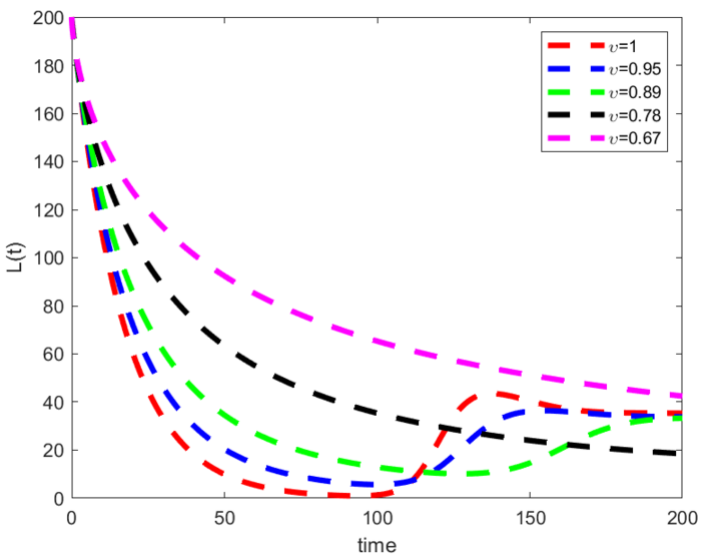
Dynamical outlook of the commulative density of the lockdown class with different fractional-order values

Considering the values in the table, we depicted the profiles of each variable under Caputo fractional derivative in Fig. 1 with the fractional-order value $\upsilon =0.67$, while Figs. 2 to 4 are the illustration and dynamical outlook of each variable with different fractional-order values. From Fig. 2(a), one can see that the susceptible class $S(t)$ shows increasing-decreasing behavior and with the lower values of *υ* the rate of decreasing starts to disappear and the rate of increasing starts becoming higher. With the same values as can be seen in Fig. 2(b), the susceptible class under lockdown $S_{L}(t)$ has also increasing-decreasing behavior with the lower values of *υ*. The decreasing rate also starts to disappear. In Fig. 3(a), the infected class $I(t)$ is virtually having the increasing-decreasing nature and with lower fractional-order values, the class totally becomes stable. In Fig. 3(b), the infected class under lockdown $I_{L}(t)$ is virtually retaining the increasing-decreasing nature, whereas the class is likely to be at stake. Figure 4 depicts the dynamical outlook of the population under lockdown $L(t)$. An interesting behavior can be noticed, one can see that there is a strongly decreasing nature in this case, this could be due to the dangers associated with the class. Under lockdown, there are many that are infected, asymptomatic, symptomatic individuals. This could affect the death rate to get higher thereby bringing the number of lockdown people to get a vehement decrease.

## Conclusions

In conclusion, we studied a system of five nonlinear fractional-order equations in the Caputo sense to examine the significance of lockdown in mitigating the spread of coronavirus. The fixed point theorems of Schauder and Banach respectively were employed to prove the existence and uniqueness of solutions of the proposed coronavirus model under lockdown. Stability analysis in the frame of Ulam–Hyers and generalized Ulam–Hyers was established. The fractional variant of the model under consideration via Caputo fractional operator has numerically been simulated via first-order convergent numerical technique called fractional Euler method. We depicted the profiles of each variable under Caputo fractional derivative with the fractional-order value $\upsilon = 0.67$. The illustration and dynamical outlook of each variable with different fractional-order values were examined.
